# Supplementary material for: Maternal morbidity profile in hospitalizations in the Unified Health System in São Paulo, Brazil: Analysis using data mining, 2014 to 2019
Source: PLoS One. 2025 Oct 22;20(10):e0323032. doi: 10.1371/journal.pone.0323032 (PMC12543157; doi:10.1371/journal.pone.0323032)
Supplement: S1 Table — (DOCX) [file pone.0323032.s001.docx]

**S1 Table – Diagnostic groups of hospitalizations adapted using the WHO classification causes of maternal death as reference**

| **Maternal ICD WHO** | | **Morbidity Study SIH/SUS** | | |
| --- | --- | --- | --- | --- |
| **Group** | **ICD** | **Group** | **Sub-Group** | **ICD** |
| 1.Pregnancy that ends in miscarriage | O001-O007 | 1.Abortion | 1a. Abortion without complications | O01**,** O02, O03.4, O03.9, O04.4, O04.9, O05.4, O05.9, O06.4, O06.9, O07.4, O07.9 |
|  |  |  | 1b.Abortion with complications | O00, O03.0, O03 .1, O03.2, O03.3, O03.5, O03.6, O03.7, O03.8, O04.0, O04 .1, O04.2, O04.3, O04.5, O04.6, O04.7, O04.8, O05.0, O05 .1, O05.2, O05.3, O05.5, O05.6, O05.7, O05.8, O06.0, O06 .1, O06.2, O06.3, O06.5, O06.6, O06.7, O06.8, O07.0, O07 .1, O07.2, O07.3, O07.5, O07.6, O07.7, O07.8, O08. |
| 2. Hypertensive causes in pregnancy, childbirth and the postpartum period | O11-O16 | 2.Hypertensive disorders | 2a. Pregnancy-specific hypertension | O11-O16 |
|  |  |  | 2b. Chronic hypertension | O10, I10, I11, I12, I13, I15 |
| 3. Obstetric haemorrhages | O20; O43.2, O44.1, 045, O46; O67; O71.0; O71.1, O71.3, O71.4, O71.7; O72 | 3.Obstetric haemorrhages |  | O20, O43.2, O44.1, 045, O46, O67, O71.0, O71.1, O71.3, O71.4, O71.7, O72, N938 e N939. |
| 4. Pregnancy-related infections | O23, O41.1, O75.3, O85, O86, O91 | 4.Infections | 4a. Pregnancy-related infections | O23, O41.1, O75.3, O85, O86, O91, N390, N110, N111, A400, A401, A402, A403, A408, A409, A410, A411, A412, A413, A414, A415, A418, A419, A46, A480, A481, A482, A483, A484, A488. |
|  |  |  | 4b. Infections not related to pregnancy | O98, A15-A19, A50-A64, A80-B09, B15- - B34, B50-B64 |
|  |  |  |  |  |

| 5. Other obstetric complications | O21.1, O21.2, O22.3, O22.5, O22.8, O22.9, O24.4, O26.6, O26.9, O71.2, O71.5, O71.6, O71.8, O71.9, O730, O731, O75.4, O75.8, O75.9, O87.1, O87.3, O87.9, O88, O90. | 5. Other obstetric complications |  | O21.1, O21.2, O22.3, O22.5, O22.8, O22.9, O26.6, O26.9, O71.2, O71.5, O71.6, O71.8, O71.9, O730, O731, O75.4, O75.8, O75.9, O87.1, O87.3, O87.9, O88, O90. |
| --- | --- | --- | --- | --- |
| 6. Unanticipated complications | O29.0, O29.1, O29.2, O29.3, O29.5, O29.6, O29.8, O29.9, O74.0, O74.1, O74.2, O74.3, O74.4, O74.6, O74.7, O74.8, O74.9, O89.0, O89.1, O89.2, O89.3, O89.5, O89.6, O89.8, O89.9. | 6. Unanticipated complications |  | O29.0, O29.1, O29.2, O29.3, O29.5, O29.6, O29.8, O29.9, O74.0, O74.1, O74.2, O74.3, O74.4, O74.6, O74.7, O74.8, O74.9, O89.0, O89.1, O89.2, O89.3, O89.5, O89.6, O89.8, O89.9. |
| 7. Non-obstetric complications | O10, O24.0, O24.2, O24.3, O24.9, O98, O99.  Indirect cause codes frequently recorded in other chapters: G00-G99, I00-I99, J00-J99, J00-K93, M00-M99, N00-N99, T82 | 7. Diabetes | 7a. Gestational diabetes | O24.4 |
|  |  |  | 7b. Non-gestational diabetes | O24.0, O24.1; O24.2, O24.3, O24.9, E10, E11, E12, E13, E14 |

|  |  | 8. Non-obstetric complications | 8a. Diseases of the respiratory system complicating pregnancy, childbirth and the postpartum period | O99.5, J00-J99 |
| --- | --- | --- | --- | --- |
|  |  |  | 8b. Diseases of the digestive system complicating pregnancy, childbirth and the postpartum period | O99.6, K00-K93 |
|  |  |  | 8c. Diseases of the circulatory system complicating pregnancy, childbirth and the postpartum period | O99.4, I00-I09, I20-I99 |
|  |  |  | 8d. Anemia complicating pregnancy, childbirth and the postpartum period | O99.0, D50-D64 |
|  |  |  | 8e. Other non-obstetric diseases complicating pregnancy, childbirth and the postpartum period | O99.1, O99.2, O99.3, O99.7, O99.8, D65-D89, E00-E09, E15-E90, F00-F99, G00-G99, L00-L99, C00-D48, H00-H95, M00-M99, N00-N10, N118, N119, N12-N38, N391, N392, N393, N394, N398, N399, N40-N930, N940-N99, Q00-Q99 |
| 8. Unknown causes | O95 | 9. Unknown causes |  | O95 |
|  |  | 10. Childbirth |  | O80-O84 |
|  |  | 11. Other obstetric conditions |  | O21.0, O21.8, O21.9, O22.0, O22.1, O22.2, O22.4, O30-O36, O40, O41.0, O41.8, O41.9, O42, O43, O440, O47, O48, O60-066, O68, O69, O75.0, O75.2, O75.5, O75.6, O75.7. |
| 9. Coinciding causes | Y85, V01–V99, W00–Y04, Y06–Y09, Y98, X85–Y04+ Y06–Y09, Y87, Y05, Y10–Y34, Y86, T65 | 12. External causes |  | ICD groups S, T, V, W, X, Y |
|  |  | 13. Others |  | ICD not classified in previous groups |
