# Supplementary figures and images for: Maternal morbidity profile in hospitalizations in the Unified Health System in São Paulo, Brazil: Analysis using data mining, 2014 to 2019
Source: PLoS One. 2025 Oct 22;20(10):e0323032. doi: 10.1371/journal.pone.0323032 (PMC12543157; doi:10.1371/journal.pone.0323032)

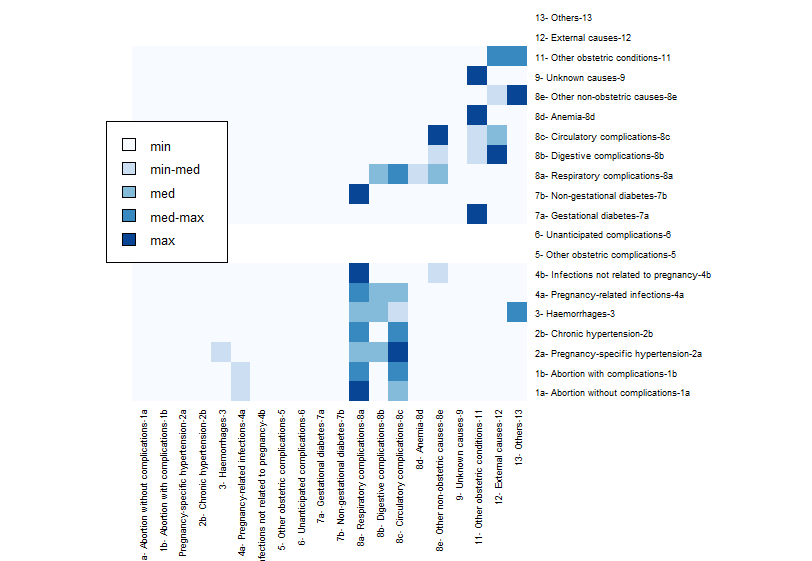

Supplement: S1 Fig — Brazil, São Paulo state, 2014–2019. (TIFF) [file pone.0323032.s002.tiff]

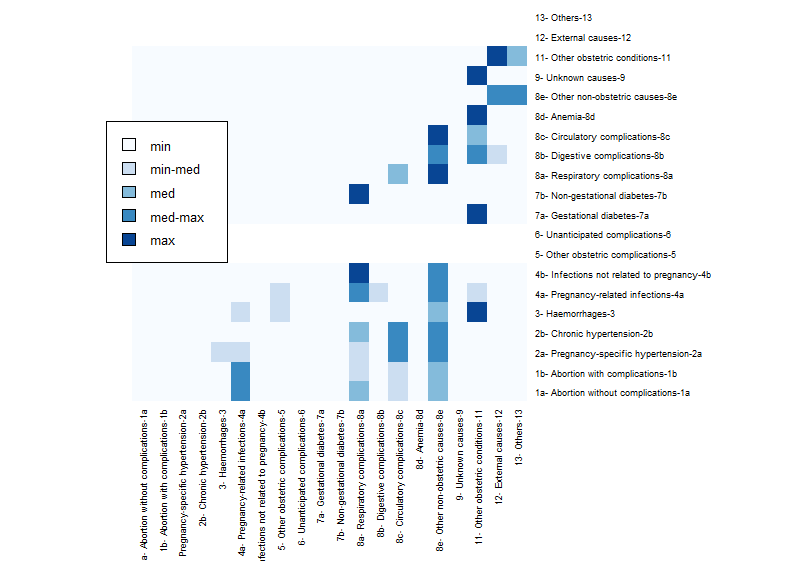

Supplement: S2 Fig — Brazil, São Paulo state, 2014–2019. (TIFF) [file pone.0323032.s003.tiff]
