# Supplementary material for: Maternal morbidity profile in hospitalizations in the Unified Health System in São Paulo, Brazil: Analysis using data mining, 2014 to 2019
Source: PLoS One. 2025 Oct 22;20(10):e0323032. doi: 10.1371/journal.pone.0323032 (PMC12543157; doi:10.1371/journal.pone.0323032)
Supplement: Attachment — Submitted filename: 2025_PONE.docx [file pone.0323032.s004.docx]

**PONE-D-25-13894, research article**

This manuscript advances the field of women's health by proposing to analyze the profile of morbidities associated with maternal death registered in the SIH/SUS, a public in hospital information system in Brazil. The study of maternal morbidity is an essential component of maternal health surveillance, contributing to improving obstetric care, as it is more frequent than maternal deaths and shares the same determinants, as stated by the authors. In addition, the manuscript uses robust, population-based data, which is often underutilized, combined to the data mining method applied to identify diagnosis groups associated with hospital discharge (alive or death).

Overall, the manuscript is interesting, well-written, presents the main results clearly and precisely, and is somewhat innovative. Below are some points for improvement to make it more suitable for publication in Plos One.

General considerations:

While the study appears to be sound, the language is unclear, making it difficult to follow. I advise the authors improve the writing quality. As an example, some parts are very literal and confusing, compromising readability.

The excerpt from the introduction “L.66 In Brazil, approximately 80% of births are publicly funded [3]...” explains well what is not quite clear in the summary, as it seems that only 80% of births are recorded. Review the writing in the summary.

Also take a look at the Methods section L.79 “...before the start of the COVID-19 pandemic”.

Or in the Discussion L.218-219, p.14 “owing to the greater risk of worsening conditions that in themselves do not pose a high risk of death and owing to the increased risk in women with other morbidities”

Moreover, some excerpts present previous evidence, including data, without adequately presenting the source/reference. Here is some of what was identified:

1) The first two sentences of the introduction (L.49-51) contain information/evidence and no references.

2) The same applies to lines 61-62.

Do not use a reference at the end of the paragraph, but at each previous piece of evidence presented. Revise.

3) Concepts on lines 124-133 requires a reference.

In the discussion, lines 221-226, p. 14 requires referencing it.

In line 214 too. And so on…

Specific considerations:

Introduction: In the first paragraph, the authors highlight the high rate of maternal mortality. However, in the 3rd paragraph they state that “maternal deaths are infrequent”. It would be interesting to revise the writing so that these ideas are clearer and do not suggest contradiction. The authors should clarify the following sections to avoid confusion.

The title, objective in the abstract and objective in the introduction of the study highlight the method (data mining). Sometimes this is more prominent than the objective of the study itself. Is this really necessary? If so, the authors will need to present in more detail why this method advances on other existing methods, in order to argue that the use of this method is also objective and part of the study problem. Also, the objective of the abstract and the introduction are very different, I suggest standardizing them.

Methods: Authors declare they have “excluded 222 hospital episodes with “death in hospital” as the reason for discharge because we could not identify deaths in the mortality database via a deterministic matching algorithm.”, but do not recall this limitation in the discussion section stating how could this affect their results. Couldn't this exclusion of 222 episodes bring an important bias to the research, since maternal death is a rare event?

Moreover, the procedure “All AIH records of the same patient identified by the algorithm were grouped and analyzed as one hospital episode.” is not very clear, because couldn't a woman have had several hospitalizations in the period studied, with different AIHs, and from different pregnancies? How were these repetitions treated? What was their frequency? Was it 1.1%? (L101, p.5?) Clarify.

“…the WHO maternal ICD groups were adapted to capture all diagnoses recorded during…” 🡪 how was the adaptation?

Results: In table 1, identify the study population in the title What would be others?

The supplementary tables are difficult to see.

Discussion: In the third paragraph, authors established differences between their results with the SIM results. But it seems like they lack pointing out how the additional information after 42^nd^ day and external causes that were included might advance in monitoring maternal morbidity and mortality….

Authors point the result of the study as follows “We also found that abortion and diabetes were associated with maternal death in the presence of other diagnoses, such as nonobstetric causes”, but do not discuss it, specifically. This could be quite relevant because it is potentially prevented.

Authors should also consider to write the implications of the results found, which aligns with the ones of Maternal Mortality causes of death, but also advances showing other results, including combinations of diseases and causes, along with the great amount of nonidentified causes…

What is the reference on the L267-270?

The first paragraph of conclusion is not the conclusion itself. I recommend to include it to the discussion section when highlighting the relevance of the study.
